# A systematic review of the effect of performance-based financing interventions on out-of-pocket expenses to improve access to, and the utilization of, maternal health services across health sectors in sub-Saharan Africa

**DOI:** 10.7189/jogh.13.04035

**Published:** 2023-04-21

**Authors:** Miriam Nkangu, Julian Little, Olumuyiwa Omonaiye, Roland Pongou, Raywat Deonandan, Robert Geneau, Sanni Yaya

**Affiliations:** 1 School of Epidemiology and Public Health, University of Ottawa, Ottawa, Canada; 2 Health Promotion Alliance Cameroon (HPAC), Yaounde, Cameroon; 3 Centre for Quality and Patient Safety Research, Institute for Health Transformation, Deakin University, Melbourne Burwood Campus, Australia; 4 Centre for Quality and Patient Safety Research -Eastern Health Partnership, Box Hill, Victoria, Australia; 5 Department of Economics, University of Ottawa, Ottawa, Canada; 6 Interdisciplinary School of Health Sciences, University of Ottawa, Ottawa, Canada; 7 Faculty of Health Science, University of Cape Town, South Africa; 8 School of International Development and Global Studies, University of Ottawa, Ottawa, Canada; 9 The George Institute for Global Health, Imperial College London, London, UK

## Abstract

**Background:**

Performance-based financing (PBF) assumes that subsidizing user fees for maternal health services to reduce out-of-pocket expenses will expand coverage and reduce inequities in access to maternal health services. It is usually associated with process changes, and the idea that increasing a facility’s resources from PBF interventions can improve the availability of equipment, drugs, and medical supplies at the facility, has an indirect effect on out-of-pocket expenses. Assessment of complex interventions such as PBF requires consideration of specific underlying assumption or theories of change. Such assessment will allow a better and broader understanding of the system’s strengths and weaknesses, where the gaps lie, whether the theory of change is sound, and will inform policy design and implementation.

**Methods:**

Following Preferred Reporting Items for Systematic Reviews and Meta-Analyses (PRISMA 2020) checklist, we performed a systematic review and a critical appraisal of selected studies using the risk-of-bias criteria developed by the Cochrane Effective Practice and Organisation of Care. We used the Grading of Recommendation and Evaluation, Development and Assessment framework for assessing the overall strength of the evidence.

**Results:**

After the abstract screening (n = 9873), we deemed 302 as relevant for full-text screening and assessed 85 studies for review eligibility. Finally, we included 17 studies in the review. We could not conduct a meta-analysis, so we report a narrative synthesis. As an add-on to an existing payment mechanism, PBF may facilitate the removal of operational barriers to enhance utilization of certain maternal health services in some contexts, especially in public facilities.

**Conclusions:**

PBF strategies may potentially decrease out-of-pocket expenses for specific maternal health services, especially in settings that have already instituted some form of user fee exemption policies on maternal health services. The implementation of PBF can be considered a potential access instrument in reducing out-of-pocket expenses to stimulate demand for maternal services. However, the implementation approaches employed will determine utilization, taking into consideration existing equitable and inequitable access characteristics which vary by context.

**Registration:**

PROSPERO CRD42020222893

Following the 1987 Bamako Initiative, whose aim was to address severe concerns in financing care in the fields of primary health care and maternal health, most sub-Saharan African countries adopted user fees as a means of financing health care, which resulted in an increase in out-of-pocket (OOP) expenses to consumers [1-6]. This policy was part of a structural adjustment program promoted by the World Bank [1,2]. However, the introduction of user fees was later found to be a major barrier in accessing essential maternal and child health services, especially among poor individuals, with potential adverse consequences of impoverishing households, eg, catastrophic payments [1-6]. Recently, there has been an increase in the momentum and international discussions on the need to introduce reforms to subsidize or abolish OOP expenses as a strategy to increase access to, and utilization of, maternal health services, especially in sub-Saharan Africa [4].

Generally, OOP expenditures are defined as the direct payments made by individuals to health care providers at the time of service utilization and are categorized into formal and informal fees [7]. These payments include fees for medications, contraceptives, laboratory tests, provider services, facility fees, and transportation expenses and exclude any prepaid payments, such as insurance fees [7-9]. Financial aspects have also been reported to be a major barrier to accessing antenatal care (ANC) and skilled birth delivery in most sub-Saharan African countries [1-6], where these financial barriers, among other factors, have contributed to high rates of maternal mortality, reaching up to 600 per 100 000 live births [10-13]. Many studies performed in Ghana, Burkina Faso, Sierra Leone, Malawi, Senegal, Kenya, and Burundi, among other countries, have shown that abolishing user fees has a substantial positive effect on the number of institutional deliveries [1,4,10-16] · In Burkina Faso and Ghana, user fee reforms were found to result in up to a 25 percentage-point increase in institutional deliveries [4]. Other studies have found inequities in outcomes, benefiting rich individuals more than poor individuals [4].

To address some of these maternal health problems, performance-based financing (PBF) is being implemented in many sub-Sahara African countries, largely focused on maternal health services [7]. PBF is a supply-side strategy that pays providers incentives based on pre-defined quality and quantity criteria [8,9,17-19]. It aims at paying providers incentives to change their behaviour (whereby they are motivated with incentives through policy changes) and stimulate demand for services by a change in cost and increase the ability of individuals to access services based on affordability [17-19]. One of the pathways through which PBF can influence the use of maternal services is the effect on OOP expenses [17-19]. For example, PBF strategies may reduce OOP expenses to enhance utilization and receive financial rewards [17-19] and, in some cases, reduce informal or unofficial fees, which are payments made in addition to official fees, also called “under-the-table” payments [8,9]. PBF assumes that “subsidizing user fees and incentivizing providers to improve financial protection by reducing OOP expenditures will increase the utilization of maternal services and reduce inequities” [8]. Within this assumption, PBF intersects with universal health coverage (UHC), defined as “expanding the coverage of health services for the general population, especially for the poorest” [8,9].

PBF is usually associated with process changes. For example, increasing a facility’s resources due to PBF interventions can improve the availability of equipment, drugs, and medical supplies at the facility, thus indirectly affecting OOP expenses regarding cost for drugs purchase within the facility compared to private pharmacy off the health facility, which may directly reduce OOP expenses related to ANC, contraceptives, and skilled birth delivery [17-20]. In some PBF designs, demand-side incentives to offset OOP expenses are incorporated as best practices to enhance effectiveness [8,17,18,21,22], and the exemption of user fees for the most vulnerable group intended to stimulate demand from this group [17,18]. However, it is also possible that PBF could increase the level of inequality in access if providers only select services or target services that are easier to reach [17,23].

Previous reviews have reported PBF interventions on OOP expenses as a secondary outcome with a focus only on consultation fees in low- and middle-income countries [20,24-27]. However, a consultation fee is only one of the items constituting OOP expenses. Previous reviews did not specify what aspects of consultation fees may include other services not related to maternal health. Through this review, we investigated the effect of PBF on each OOP expenses for maternal services as defined above, including both demand-side (implemented to enhance PBF) and supply-side PBF strategies. We build on the above PBF assumption on subsidizing user fees to reduce OOP expenses and apply Andersen et al.’s contextual and enabling characteristics of the behavioural model on health care utilization to discuss the outcomes, using their six dimensions of access (Table S1 in the **Online Supplementary Document**) [28]. As per the protocol [29], the rationale for applying this model [28] was to understand the interplay of the contextual and individual characteristics and how PBF, as a mechanism to stimulate behaviour change, interacts with this to increase access and utilization of maternal services and improve equity.

In most sub-Saharan African countries, health care is largely delivered in both public and private sectors, and PBF interventions are mostly implemented across all health sectors. Additionally, OOP expenses for maternal health services in the public sector differ from those in the private sector, where higher OOP expenses in accessing maternal services have been reported [1-6,19]. Given the high OOP expenses that impede access to maternal services in sub-Saharan Africa, evidence is lacking on how PBF mediates OOP expenses to improve access to ANC, skilled birth delivery, and family planning across the health sectors.

Assessment of complex interventions such as PBF requires a review of the approaches aimed at assessing specific underlying assumptions or theories of change. This will allow a better and broader understanding of the system’s strengths and weaknesses, where the gaps lie, and whether the assumption or theory of change is sound, in order to inform policy design and implementation [19-28,30,31]. It also helps with identifying missing links or gaps in the process to further inform programs and intervention designs [19-28,30,31]. Such understanding can inform country-level policies for PBF on ANC, skilled birth delivery, and family planning. This review aligns with the growing call for UHC and for addressing equity in access to maternal health services, as well as the global action for health promotion, which calls for sustainable financing [32]. Thus, we sought to assess the effect of PBF interventions on OOP expenses as a mediating factor toward improving access to, and the utilization of ANC, skilled birth delivery, and family planning. This review was guided by the following questions: 1) What are the PBF supply-side and/or demand-side interventions implemented or identified toward reducing OOP expenses for ANC, skilled birth delivery, and family planning in sub-Saharan Africa? 2) What is the effect of PBF interventions on OOP expenses in improving access to and the utilization of ANC, skilled birth delivery, and family planning and across health sectors in sub-Saharan Africa? We used the population/intervention/comparison/outcome (PICO) framework in formulating the research questions [29].

## METHODS

### Protocol and registration

We registered the protocol (PROSPERO CRD42020222893) and published it with BMC Systematic Reviews [29] and reported this review using the Preferred Reporting Items for Systematic Reviews and Meta-Analyses (PRISMA 2020) reporting guidelines [33,34]. We performed a critical appraisal of selected studies using the risk-of-bias criteria developed by the Cochrane Effective Practice and Organization of Care (EPOC) Group for randomized and non-randomized studies [35]. We used the Grading of Recommendation and Evaluation, Development and Assessment (GRADE) tool to assess the overall strength of the evidence with respect to effect estimates [36-38]. Additionally, two reviewers (MN, OO) independently assessed the quality of systematic reviews using the Assessing the Methodological Quality of Systematic Reviews (AMSTAR) tool [39].

### Search strategy, selection criteria, and data extraction

We designed our search terms in consultation with an information specialist, after which we combined the search terms for PBF and sub-Saharan Africa. We first developed the search criteria in Ovid (MEDLINE) and then adapted the search strategy to the following databases: EMBASE, CINAHL, Cochrane Library, and CABI. We also searched the World Bank e-library (for PBF impact evaluations), Department for International Development research output database, and ELDIS development database. We did not set a publication year limit, and the last search was conducted on 30 December 2022. We screened the reference lists of all the included papers for additional studies. Additionally, we searched clinical trial registries to identify completed and/or in-progress studies and hand-searched relevant study references. The full electronic search strategy for all databases is available in Table S2 in the **Online Supplementary Document**.

To be included in the review, each study had to meet the following criteria: 1) the intervention must be a PBF strategy and may include a demand-side intervention implemented as part of a PBF program, 2) the intervention must include consideration of aspects of OOP expenses aimed at either directly or indirectly enhancing access and utilization, 3) the study results must quantitatively report on the impact on access to and the utilization of ANC, skilled birth delivery, and family planning, irrespective of the direction of the effect. We excluded papers that reported the outcomes, but did not assess or report on any pathway of PBF intervention in reducing OOP expenses. We included randomized controlled trials (RCTs) and quasi-experimental studies conducted in sub-Saharan Africa and published in English or French. We also assessed the effect of PBF across the different health sectors. For the outcome, this included any reported PBF interventions on OOP expenses for ANC, skilled birth delivery, and family planning or any reported (or observed) changes in ANC, skilled birth delivery, and family planning attributed directly or indirectly to PBF effect on OOP changes and across the health sector. Finally, we excluded commentaries, perspectives, expert opinions, conference proceedings, and editorials.

### Study selection and data extraction process

Two reviewers (MN, OO) screened the studies and assessed them for eligibility. We exported the search results to Endnote for duplication removal and referencing and to Covidence [40] for additional duplicate removal and independent screening. MN and OO conducted the initial title and abstract screening, developing the inclusion and exclusion criteria through calibration on a small number of studies to establish clarity. We resolved any disagreements through consensus, consulting additional reviewers (SY and JL) if consensus was not achieved. We retained all studies reporting an intervention using PBF and our outcomes. When we could not retrieve full-text articles could, we contacted the authors. We excluded studies that only reported PBF interventions toward ANC, skilled birth delivery, and family planning without reporting any intervention effect or policy change on OOP expenses. We also excluded all studies that were not performed within sub-Saharan Africa.

Two researchers (MN, OO) independently extracted the data from the included studies using a data extraction form adapted from EPOC [41]. We resolved any disagreements through discussion, consulting other reviewers (JL and SY) when consensus was not achieved. We contacted the authors of the included studies (twice if necessary) when the extraction was difficult or unclear or when data were missing. We extracted data on the year of PBF implementation, health sectors, and health system financing mechanisms in place before PBF, with a specific focus on the health system policies that were implemented on maternal health before PBF. We then extracted methodological and population-level data on the study design and objectives, population of interest, study outcomes, and any equity considerations. Additionally, we extracted data on access measures adapting the six dimensions of access as described by Andersen et al. [28]. For the intervention, we extracted data on any reported OOP expenses related to medication costs (specifically for those during ANC), contraception fees, skilled birth delivery, laboratory tests, and ANC fees.

### Assessment of risk of bias in individual studies

We used the EPOC risk-of-bias tool to assess the quality of the methodology used for RCTs, controlled before-and-after studies (CBA), and interrupted time series analyses (ITS) [34]. We applied this tool according to its criteria for the three study designs, based on which they are categorized as “high”, “low”, and “unclear” [35]. Two reviewers (MN and OO) independently assessed risk of bias, resolving any disagreements through consensus.

### Summary measures of effects

We used the GRADE tool for the summary of findings [36]. Only studies that met the following criteria were included in the appraisal: RCTs, CBAs, and ITS with a predefined shape that clearly states the pre-intervention period and clearly defines the point of analysis as the point of intervention [35]. We only narratively described the studies that did not meet these criteria. We extracted post-intervention effect estimates for RCTs, CBAs, and recorded changes in the level and slope for ITS. Difference-in-differences analysis was the most used analysis across all study designs, adjusting for covariates and potential confounders. Most of the studies generated relative effects reported as regression betas. Some of them did not report confidence intervals, or in some cases, a standard deviation. We recalculated the regression betas for eight studies (two RCTs and six CBAs) using the approach described in the Cochrane review by Diaconu et al. [25]. Thus, we divided the effect estimate of the beta by the baseline control group mean and multiplied it by 100 to obtain the relative percentage change for the outcome attributable to the intervention [25]. We did not re-analyse the data for the two ITS and did not extract data for results presented graphically. We then appraised the analysis for each study included in the summary of findings table to ensure that the studies are adjusted for clustering and controlled for potential confounders.

Poor reporting of data prevented adequate assessment of statistical heterogeneity and made it impossible to perform a robust meta-analysis. We also noted this as a gap in the way measures of precision were reported which limited the possibility of pooling the data for meta-analysis. Therefore, we reported the results as a narrative synthesis using the synthesis without meta-analysis guidelines (SWiM) [42] and excluded less robust studies in the summary of findings table.

To be eligible for the GRADE synthesis, each study must have measured OOP expenses in one of the following ways and reported the effect estimates: the probability of paying for OOP expenses during skilled birth delivery (or the percentage of women who paid for delivery), the probability of paying for any ANC visits, or the probability of paying for family planning. Alternatively, each study must have reported any change in user fees or presented results that compare any additional demand-side subsidies to offset OOP expenses vs a control group or reported results or effects of any policy change because of PBF on OOP expenses for ANC, skilled birth delivery and family planning.

We carried out a subgroup assessment for each outcome if the authors reported and disaggregated results that compared the additional incentives or strategies to offset OOP expenses on the outcome and by wealth quintile. This was achieved by extracting data for each outcome as reported in the study according to specific intervention groups based on the additional incentives and/or wealth categories. We did a sensitivity analysis based on the study design by reporting the results for each outcome assessed using an RCT [25]. Thereafter, we assessed the certainty of the evidence using the EPOC group worksheet criteria (high, moderate, low, or very low, as well as the GRADE consideration for the risk of bias, inconsistency of the results, imprecision, indirectness, and publication bias) [35-38]. In the absence of meta-analyses, we used the EPOC table recommended for presenting findings without a meta-analysis, assessed the level of certainty to present the overall direction of effect of the intervention, and provided justifications for the assessed outcome [35-38]. GRADE assessments were conducted by two independent reviewers (MN and OO), and disagreements were resolved through discussions and consultation with SY and JL.

## RESULTS

**Figure 1:**
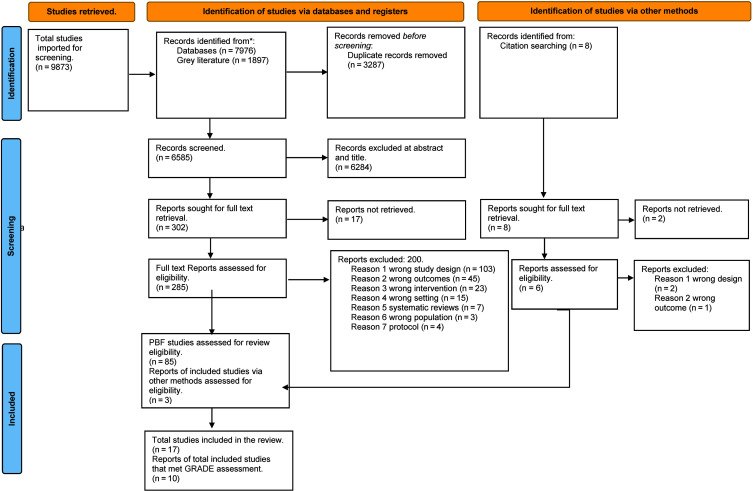
PRISMA flow diagram, adapted from [34].

We screened a total of 9873 abstracts, 302 of which were relevant for full-text screening. Seventeen studies could not be retrieved. A further 200 were excluded, and 85 were PBF studies eligible for additional screening considering the initial objective of updating some of the outcomes of an earlier Cochrane review published in 2012. However, the Cochrane review was updated in the meantime [25], so we included 17 studies which were within the focus of this review (see PRISMA flow diagram in **Figure 1** and list of excluded studies in Table S3 in the **Online Supplementary Document**.

**Figure 2:**
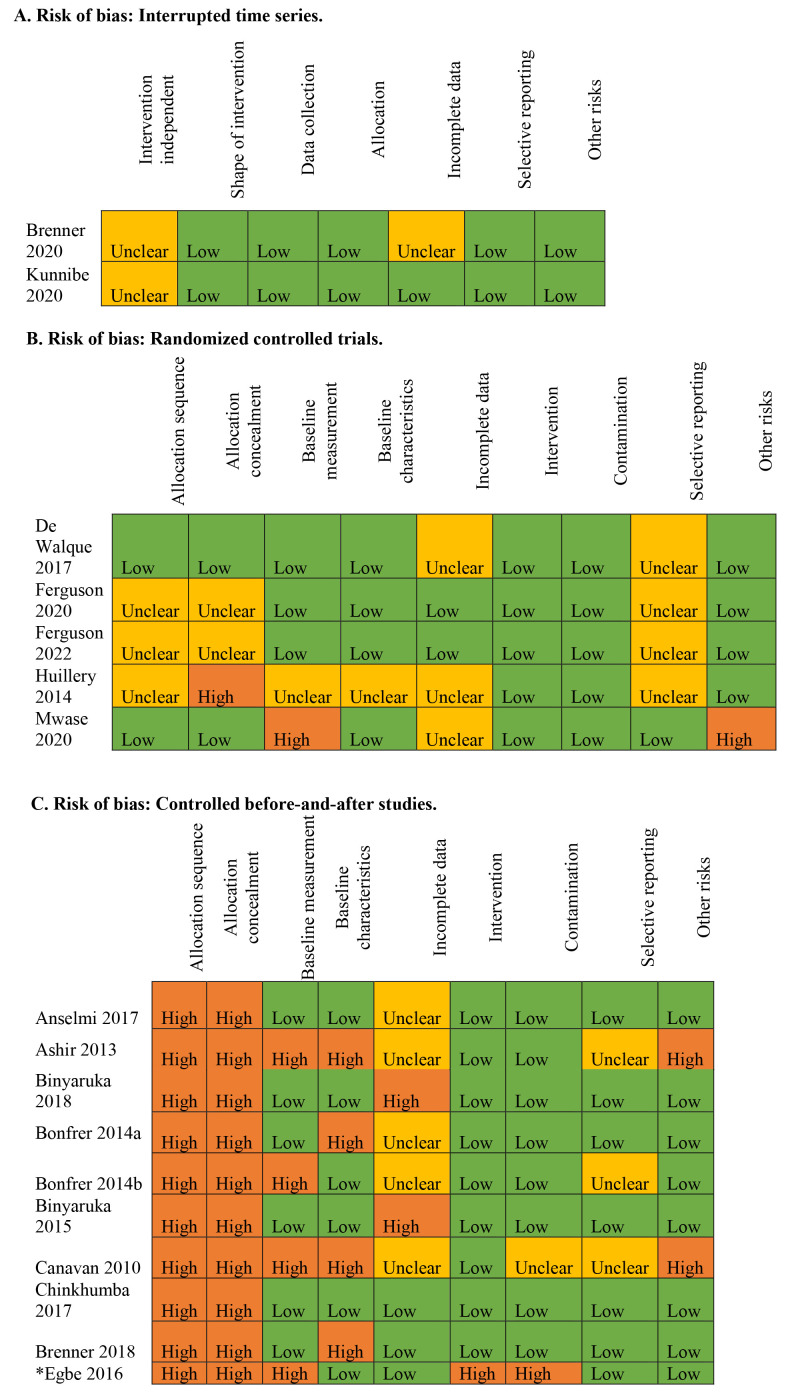
Risk of bias assessment for the 16 included studies. **Panel A.** Interrupted time series. **Panel B.** Randomized controlled trials. **Panel C.** Controlled before-and-after studies. *Cross-sectional study.

Most studies included a control group, some used matched controls, some were evaluated at the subnational and national level, and most were evaluated at the district and facility level. Nine of the studies were CBA studies [17,19,21,43-48], two ITS [49,50], four RCTs [22,51-53], one a cluster-RCT [54], and another a cross-sectional study [55] (**Table 1**). The studies were conducted in Burkina Faso, Burundi, Cameroon, the Democratic Republic of Congo, Gambia, Malawi, Nigeria, and Tanzania. PBF was implemented between 2006 and 2012, and most were either pilot studies or used data from the pilot studies. Most countries deliver care in both the public and private sectors (**Table 2**), and OOP expenses varied by sector and were reported to be higher in the private than the public sector, but the results were generally not reported in a disaggregated form. Four studies (from Malawi, Gambia, and Nigeria) combined demand-side strategies (conditional cash transfers) with PBF to offset OOP expenses [21,22,46,47]. Some incentivized traditional birth attendants for referrals and outreach programs and the use of home visits to enhance access [21,22,55]. The study durations ranged from six to 57 months. Some studies adjusted for clustering at the level of the facility (even when the allocation was not done at the facility level). According to the risk of bias assessment (**Figure 2** and in Table S4 in the **Online Supplementary Document**), most CBA were of low quality mostly due to lack of randomization and allocation concealment. Some of the RCTs had high risk of bias, mostly in allocation concealment, and it was unclear how incomplete outcome data were addressed.

**Table 1:** General methodological characteristics of PBF-included studies.

Author, year (country)	Study aims	Characteristics of study population	Facility type/health settings/timeline measurement time points and follow-up	Relevant outcomes*	Data source/data analysis type	Equity consideration
**Randomized controlled trials and cluster RCT**						
De Walque et al., 2017 (Cameroon) [51]	Impact of PBF on maternal and child health service coverage and quality.	Women, children, and health providers survey 6275 women.	26 districts; 14 included in the analysis, 259 facilities, 164 public, 48 confessional, 33 private for-profit and para-public.	ANC, skilled birth delivery, family planning.	Household, facility, and exit surveys. Difference-in-difference.	Equity consideration in the PBF design (geographic accessibility, poverty, and population density).
Ferguson et al., 2020 (Gambia) [22]	Assessed factors contributing to the impact of incentivising activities related to skilled birth delivery uptake and the causes of delays in accessing skilled birth delivery.	2400 households. Head of households and women with children under five years.	Facility types not mentioned. Three regions, serving 800 communities; 22 health centres, two hospitals, 19 health facilities (three-intervention arm vs control).	Six indicators related to skilled birth delivery.	Household surveys/multivariable linear regression models.	Equity consideration in data analysis on ethnicity.
Ferguson et al., 2022 (Gambia) [53]	Assess impact of PBF interventions on uptake of targeted services and behaviours.	Households with women above 15 years and a child under five years. 20 households across 114 enumerated areas.	19 health centres and their catchment areas. 5927 household surveys: 1939 conducted at baseline, 1951 at midline, and 2037 at endline. October 2014 to June 2018.	Attending early ANC; three additional ANC visits following a timely first visit; skilled delivery at a health facility.	Household surveys. Multivariable linear regression models.	Equity consideration in the design using CCT to address barriers in access.
Mwase et al., 2020 (Burkina Faso) [54]	Examined PBF effects on maternal health service utilization and defined equity as equal access given equal need, with access measured as reported service use and need.	Women aged 15-49.	Total 24 districts, 416 facilities in intervention districts and 117 in the control districts. Participants – component 1; INT = 4932 CTL = 1439. Component 2; INT PBF1 = 1099; PBF2 = 983; PBF3 = 1010. Comparison PBF1 = 368, PBF4 = 605. Study period 2013- 2017. C-RCT nested within CBA.	Institutional deliveries, ANC visit in first trimester and at least 4 ANCs, family planning.	Survey-self reported. Difference-in-difference analysis.	Consideration of equity in both PBF design and analysis.
Huillery et al., 2014 (Democratic Republic of Congo) [52]	Assessed the effect of PBF on user fees, health worker and work-related stress, utilization of services and health status.	332 health workers, 1014 patients, household surveys 1708.	One district, 96 health areas, Admin data 152 facilities assigned into two payment groups. Survey 123 facilities in 87 health areas; 44 from PBF payment group and 43 from fixed payment group. Study period: 2009-2013.	Access-user fee, birth delivery, prenatal visits, postnatal visits.	Household and facility surveys. Difference-in-difference analysis.	Equity consideration on place-urban and rural in analysis.
**Controlled before-and-after studies**						
Anselmi et al, 2017 (Tanzania) [19]	Test the causal pathways through which PBF may influence the utilisation of maternal health services.	3000 women who have delivered: 200 health workers.	Public and private, 11 districts; 150 health facilities (75 in each arm). Seven intervention, four comparison districts, 13 mo.	Utilization of skilled birth delivery, uptake of antimalarial drugs.	Surveys/difference-in-difference analysis.	Not defined or mentioned
Ashir et al., 2013 (Nigeria) [21]	Role of demand-side PBF scheme in improving utilization of maternal services.	Women and children under five. ANC four visits, INT = 192 women, CTL = 59. Skilled delivery INT = 63 women; CTL = 20.	Public: eight community areas (six intervention, two control), six months.	Utilization of ANC, skilled birth delivery& immunization.	Administrative data/ descriptive statistics/ANOVA.	Study targeted disadvantaged groups in terms of age, poverty, education, parity.
Binyaruka et al., 2018; (Tanzania) [17]	Examined the heterogeneity of P4P effects on service utilisation across population subgroups.	3000 women who have delivered 12 months prior to survey.	11 districts (seven intervention, four control),75 facilities intervention and similar number for control, 13 months.	Institutional deliveries and uptake of two doses of (IPT2) for malaria during antenatal care.	Surveys/difference-in-difference model.	Equity considerations in the study assessment across population groups and income group.
Bonfrer et al., 2014 (Burundi) [43]	Effect of PBF on the utilization and quality of health care services.	Household survey conducted in 2006 (n = 500), 2008 (n = 300 PBF, 1050 non-PBF), 2010(n = 1350 PBF.	Nine provinces, 700 facilities. Study period: 2006-2010.	ANC, institutional deliveries, pregnant women fully immunized, use of modern family planning.	Household surveys/difference-in-difference model.	Equity consideration in the study selection criteria & assessment (income & presence of private for-profit health facilities.
Bonfrer et al., 2014 (Burundi) [44]	Extended previous work covering all provinces and using DHS data effects on the quantity of child and maternal care use and its quality during ANC visits.	4916 women (survey focus on birth from 2005-2010).	Public and private. 17 provinces; 700 facilities. Study period: August 2010-January 2011.	Antenatal care visits, institutional deliveries (in public or private).	DHS data /difference-in-difference analysis.	Equity consideration in the study assessment for income groups, private and public facilities.
Binyaruka et al., 2015 (Tanzania) [45]	Effect of a P4P scheme implemented at scale in one region on quality and utilisation of targeted and non-targeted services, user costs and equity.	3000 women who have delivered 12 months prior to the survey.	Public and private. 11 districts (seven intervention, four control), 75 facilities intervention and similar number for control. Survey conducted in 2012 and 2013 (13 months).	Antenatal care, user cost, long-term contraceptives, skilled delivery.	Household, exit and facility survey/difference-in-difference analysis.	Equity consideration in study assessment across income groups.
Brenner et al., 2018 (Malawi) [47]	Examine the impact of RBF scheme on effective coverage for facility-based obstetric care services.	Survey user sample 5509 women; obstetric sample 383 women.	Facility sample 32 facilities; 25 interventions and nine control. Study period: April 2013 to July 2015.	Facility-based obstetric care.	Household questionnaires and observation checklist. Difference-in-difference analysis.	Equity considerations in analysis on household socioeconomic status.
Chinkhumba et al.,2017 (Malawi) [46]	The impact of RBF on household costs and time to seek care for women with pregnancy-related complications.	2219 women.	Public and private facilities,17 public and after one year additional 11 facilities, six privates non for profit including one mission and five public. Study period: May 2013 to July 2015.	Skilled birth delivery.	Household surveys. Generalized linear model.	Equity consideration in assessment of outcome.
Canavan 2010 (Tanzania) [48]	Compared mission faith-based health facilities with P4P vs public facilities without P4P.	Health centre deliveries and ANC visits from 2006-2008.	Public and private facilities Targeted only private – five dioceses.	Institutional deliveries of 10 per 1000 (hospitals) and 20 per 1000 (HCs and dispensaries).	Surveys and data from health information system/descriptive analysis.	Insufficient information.
**Interrupted time series analysis**						
Kuunibe et al., 2020 (Burkina Faso) [49]	To assess impact of PBF and user fee exemption on MCH services.	Facility data on utilization for antenatal care and skilled delivery and family planning.	838 facilities (524 intervention, 314 controls) Study period: January 2013 to September 2017 (57 months).	ANC and skilled birth delivery and family planning.	HMIS data/segmented regression analysis (interrupted time series analysis).	Equity consideration in PBF design.
Brenner et al., 2020 (Malawi) [50]	To explore whether patterns of change reflected by PBF effects on selected service indicators differed between health centres and hospitals.	31 data points-no further information.	17 intervention and 17 control facilities (all public), five hospitals and 12 health centres across 8 districts. Data collected for 31 months.	Antenatal visits, skilled birth delivery, number attending family planning counselled on contraceptive options.	Baseline data used DHIS and endline data collected primary data. Interrupted time series analysis.	Insufficient information.
**Cross-sectional studies**						
Egbe et al., 2016 (Cameroon) [55]	To estimate the effect of PBF home visits on the use of modern methods of contraception by women of childbearing age in the Kumbo East Health District.	Women of childbearing aged 15-49 in the study district.	552 surveys; 226 in each group; 262 responded from intervention group and 221 from control group. Data was collected from 1 February to 31 May 2015.	Modern family planning contraceptive.	27-item survey/ANOVA and binary logistic regression.	Equity considerations in PBF design using home visits and analysis across socio-demographic factors.

ANC – antenatal care, HMIS – health management information system, DHIS – district health information system, DHS – demographic and health survey, ANOVA – analysis of variance, CCT – conditional cash transfer, PBF – performance-based financing, MCH – maternal child health, CBA – controlled before-and-after study, CTL – control, INT – intervention, P4P – pay-for-performance

*Outcomes column is only for the outcomes of interest to this review and not necessarily for all the outcomes studied by each paper.

**Table 2:** Health system characteristics of PBF-included studies including health financing mechanism before PBF, pathways of OOP expenses and overall findings

Author, year	PBF intervention/y of implementation	Health care delivery sectors	Health financing mechanisms for RMNCH before PBF	PBF intervention on OOP items (directly or indirectly), demand or supply side strategies	Main study findings
**Tanzania**					
Anselmi et al, 2017 [19]	Financial incentives to health facilities, districts and regional. conditional on performance, quality and quantity of services. PBF implemented in 2011.	Public and private sector.	Women are supposed to be exempted from payment during delivery in public facilities–not enforced effectively before PBF.	Direct – PBF induced effect on providers enforced efforts on exemptions of OOP expenses to attract women to facilities for skilled delivery – Indirect-Reduced probability of paying out-of-pocket for institutional delivery	PBF increased supervision visit which had an effect on the uptake of two doses of antimalarial during ANC visits. Reduction in proportion of women who paid for skilled birth delivery in general with more impact on public facilities. Increased utilization of SBD by 48% & in public facilities by 78%. Increased skilled delivery in rural vs urban women
Binyaruka et al., 2018. [17]	Performance incentives targeted at facilities for staff, demand creation initiatives and performance improvement, districts, and regional managers.	Mentioned of public facilities and private pharmacies.	User fee exemption policy – not enforced.	Indirect – reduced chance of paying user fee and increased availability of drugs and supplies which reduced cost of purchasing drugs from private pharmacy. Direct – reduction and increase adherence on user fee exemption for delivery especially in public facilities .	Increased adherence on user fee, significant increase in the rate of institutional deliveries among women in poorest and in middle wealth status households, but not among women in least poor households. PBF effect on institutional deliveries was significantly higher among women in rural districts compared to women in urban districts.
Binyaruka et al., 2015 [45]	Financial payments to health facilities, district and regional health managers as a bonus based on achievement of targets relating to maternal and child health care.	Public and private.	User costs for institutional delivery should be free at government facilities, although user fee exemptions are not well enforced.	Direct – PBF resulted in greater enforcement of adherence to the exemption policy, (free care) in public facilities which was measured as the share of patients paying OOP or giving gifts to service providers.	Increased institutional deliveries and the provisions of anti-malarial during antenatal visits. Positive effect on overall coverage for ANC but no effect on proportion of women having 4 or more ANC. P4P was associated with reduction in those paying OOP for deliveries but there was no evidence of an effect on the average amount paid.
Canavan, 2010 [48]	Fixed amount paid to health mission health facilities for five targeted services, to strengthen Diocesan Health Services in improving access, quality of services and organizational performance. Implemented in 2006 (CORDAID).	Public and private sectors.	Exemption of user fee is in theory as advocated by MOH–free treatment for all pregnant women, some faith based apply the policy exemption for the poor, no equity funds, or Community health funds at faith based. user fees generally expensive in faith based compared to public facilities.	Insufficient information.	PBF facilities not performing well for both institutional deliveries and ANC from 2005-2007.
**Nigeria**					
Ashir et al., 2013 [21]	Focused on demand side PBF scheme, providing cash incentives to women based on performance indicators for ANC & Skilled birth delivery, community approach not specifically targeting poor women. PBF was implemented in 2011.	Public and private but no private health facilities in the study state.	Public health facilities receive free drugs, medical supplies.	Direct demand PBF – cash incentives to women for ANC1, ANC4, and skilled birth delivery-including transportation to offset OOP expenses but not specifically for poor-universal approach.	Demand-side PBF led to an increased in utilization of up to 4 ANC visits and the use of skilled birth delivery in intervention compared to control.
**Burundi**					
Bonfrer et al., 2014 [43]	Facilities receive payments based on the quantity and quality of health services provided.PBF implemented in 2006	Public and private.	Seven months before PBF, user fees for deliveries, caesarean sections, and care for children under age five were removed at public health care facilities, throughout the country.	Direct – to replace lost income from eliminated user fees, facilities received payments from the government for the services provided for free. Ministry of health incorporated the payments for MNCH services into PBF scheme.	Increased proportion of institutional deliveries, use of modern family planning and increased overall quality score for health facility, no change in quality of care reported by patient & no differential effect across socio-economic groups.
Bonfrer et al. ,2014 [44]	Health care facilities received payments based on the quantity and quality of services provided. PBF implemented in 2006.	Public and private.	Seven months before PBF, user fees for deliveries, caesarean sections, and care for children under age five were removed at public health care facilities, throughout the country.	Direct – government converted income loss from user fees into PBF scheme and incorporated into the PBF program.	Increased in institutional deliveries especially amongst the better off but no effect on the poor. No effect on place of residence or facility type.
**Malawi**					
Brenner et al., 2020 [50]	Service delivery integrated performance-based incentive (SSDI-PBI). PBF implemented in 2014.	Public/private but PBF was implemented only in public facilities.	Essential health package meant to be free at point of care for public and private non-profit facilities contracted by MOH, but evidence indicates not effectively available subjecting clients to some substantial OOP expenses.	Insufficient information.	First trimester ANC showed positive trends in facilities and hospitals. Skilled birth delivery- no change in either facility type, or family planning counselling. In both health centres and hospitals, positive PBI effects on antenatal care included resilience against interrupted supply chains and improvements in attendance rates.
Chinkhumba et al., 2017 [46]	P4P incentives includes both supply and demand-side conditional financial incentives. RBF for MCH implemented 2013-focused only on obstetric emergency care delivery.	Public/ private.	Provision of free emergency obstetric care at all public health facilities and selected private not-for-profit facilities contracted by the government. Evidence indicates medical costs are still shifted towards patients due to stock outs of drugs and other items needed for surgery.	Demand side incentives paid to pregnant women irrespective of socio-economic status-cash transfer average US$10.50 per woman consist of a flat sum and a variable portion in relation to distance travelled and hospital stay 48 hours postpartum.	Time to seek care decreased by 34.2% at endline in PBF areas relative to non-PBF areas. No substantial change in household costs observed. PBF induced quality improvements, prompting faster decisions for obstetric care seeking at household level. Observed substantial reduction in indirect cost for households benefitting from CCT.
Brenner et al., 2018 [47]	To enhanced obstetric care provision through a combination of PBF payments to facilities and district health managers and demand side mechanism conditional cash transfers to pregnant women in the catchment areas.	Public/private sector.	Obstetric care is free through public and contracted not-for-profit health facilities, however, 75% of pregnant women do not receive satisfactory emergency obstetric care.	Demand side PBF – provision of conditional cash transfers within the PBF program provided upfront to women to offset transportation, basic childbirth items, and stay in maternal waiting home and average opportunity cost for poor patient or family during a 48-hour postpartum facility stay.	Improved coverage for pregnant women with facility-based obstetric care after a two-year implementation.
**Cameroon**					
De Walque et al., 2017 [51]	Financial Incentives rewarded to facilities based on verified quantity and quality indicators of service provision. PBF implemented in 2012	Public and private.	OOP expenses. Government subsidized delivery kit (led to a reduction in the cost of delivery kit in public facilities) within the period that PBF was introduced.	Direct laboratory and x-rays fees declined by (US$2.38), Indirect change in official provider fees for antenatal care was lower in the PBF group (US$1.68) compared to control, unofficial provider fees reduced by (US$3.64) compared to control.	PBF did not impact on ANC and skilled birth delivery, however, it had positive effect on family planning based on facility data. OOP health expenditures decreased for households in the PBF arm, including unofficial payments and this decrease in revenue did not come at the cost of process quality.
Egbe et al., 2016 [55]	PBF trained nurses to provide women education in their homes about contraception and prevention of sexually transmitted infections.	Only mentioned of public sector.	Did not clearly mentioned and provided insufficient information.	Condoms free and available in public pro pharmacies. Other contraceptive methods subsidized and available in other public facilities. Average cost of contraceptives US$2.63.	PBF home visits let to a significant increase in utilization of modern contraceptive methods in the area with PBF home visits relative to the control areas.
**Gambia**					
Ferguson et al., 2020 [22]	PBF incentivized the provision of specified maternal and child nutrition and health services, including facility-based delivery by a skilled practitioner, as well as incentivizing service quality and a conditional cash transfer (CCT) program for attending ANCs and a community-based intervention package.	Not mentioned.	Not mentioned of pre-existing financing mechanisms for maternal services.	Demand side PBF – women enrolled in a conditional cash transfer program for attending first ANC within 12 weeks and three additional ANCs and a community-based intervention package including transportation to offset OOP expenses.	No significant change in skilled delivery within the 18 months, however, the PBF scheme increased proportion of women referred to health facilities for skilled birth delivery, increased proportion of women accompanied and transported to the facility.
Ferguson et al., 2022 [53]	Facility intervention package included payment for maternal and child nutrition services and conditional cash transfer to individual women for timely use of ANC. PBF implemented 2013.	Not mentioned.	ANC and delivery services was free, but some cost associated with transportation and food reported as barriers.	Demand side – two conditional cash transfers of US$3.50 each for attending ANC within the first 12 weeks and for attending an additional three ANC visit.	No change in antenatal care, a 7%-point change in skilled birth delivery.
**Democratic Republic of Congo**					
Huillery et al, 2014 [52]	Payment was only conditional on the number of patients for some pre-determined services. less stringent on quality due to context related aspects. PBF implemented in 2010	Did not mentioned.	Did not mentioned any pre-existing financing mechanism for maternal services.	Reduction of user fees for some targeted services by health workers, medication.	No impact on use of modern family planning. Reduced OOP cost on targeted services especially facilities with high cost but PBF efforts made by health workers did not lead to change in utilization of services in the population.
**Burkina Faso**					
Kuunibe et al., 2020 [49]	Incentives rewarded to facilities based on verified quantity and quality indicators of service provision. PBF implemented in 2011	Health care delivery mostly organized via public sector.	User fee exemption for skilled birth delivery before PBF.	Two years after PBF scaled up, in 2016 government introduced nationwide free health for maternal health services.	Before free health services, PBF increased in trends of ANC 0.4% and 0.1% for family planning, no effect on SBD. With free health services, PBF did not have any effect on services and a decreased of 0.3% family planning. Moderate increase in use of ANC and family planning but no change in skilled birth delivery. However, with introduction of the nationwide free services, there was no further increase in this service provisions.
Mwase et al 2020 [54]	Four different models, three of which included an equity intervention targeting specifically the ultra-poor Health facilities were rewarded based on defined health service indicators using a case-based payment system, adjusted for quality of care after verification. PBF implemented in 2011 and equity measures combined in 2014.	Did not mentioned.	Removal of user fee for ANC in 2002 and 80% removal fees for skilled birth delivery in 2007.	Services provided to ultra-poor reimbursed at a higher unit price to compensate for lost revenue from user fees and to incentivized providers to reach out to poor. In 2016 government introduced nationwide free health for maternal health services. MOH adjusted PBF prices by removing additional equity payments due to complete removal of user fees.	PBF produced modest changes compared to status quo, the implementation of equity interventions did not generate additional benefits compared to PBF alone, neither for women in general nor for the poorest women specifically.

RMNCH – reproductive maternal newborn child health, SBD – skilled birth delivery, P4P – pay-for-performance, OOP – out-of-pocket, CCT – conditional cash transfer

*Findings column is only for the outcomes of interest to the review and not necessarily all the outcomes studied by each paper.

### Characteristics of PBF intervention strategies, including setting and health policies in place

Most of the PBF interventions were compared against standard care; in some cases, PBF schemes were compared with nationwide free services. Of the eight countries included in the review (**Table 2**), Tanzania reported an unenforced exemption policy in public facilities before PBF [17,19,48]. Following PBF, providers re-enforced the user fee exemption policy to reduce OOP expenses [19,45]. The two CBA studies reported a significant reduction in OOP expenses and increased utilization of institutional deliveries [19,45]. One study further examined this mediating effect of OOP expenses on skilled birth delivery in Tanzania and across sectors and reported an increase of 48% in institutional deliveries in private and 78% in public facilities [19]. Burundi reported a user fee exemption policy for deliveries and caesarean sections before PBF, as well as some potential constraints on transportation costs for poor individuals [43,44]. The government replaced the income loss from abolishing user fees (six months before PBF) by incorporating payments of maternal and child services to the PBF program [43,44]. Before PBF, Burkina Faso had a user fee exemption policy for ANC, as well as 80% exemption of user fees for delivery care and full exemption for very poor individuals [49,54]. After its implementation, Burkina Faso introduced a nationwide user fee policy for pregnant and lactating women [49,54].

Demand-side intervention such as conditional cash transfer was reported in Nigeria, Malawi, and Gambia [21,22,46,47,53]. Women were provided cash incentives for either ANC and/or skilled birth delivery and, in some cases, transportation costs to offset some elements of OOP expenses to enhance the utilization of ANC and skilled birth delivery. In Malawi, before PBF, all primary health care services included in the essential health package were intended to be free at the point of care in both the public and private sectors [50]. However, evidence indicates that such services are not effectively available, subjecting women to substantial OOP expenses [50]. PBF did not result in any substantial effect on household costs, but an observed substantial reduction in indirect costs for households benefitting from conditional cash transfers was reported, which suggests PBF can potentially reduce overall burdens on households [46].

Notably, Cameroon, the Democratic Republic of Congo, Nigeria, and Gambia did not report any user fee exemption policies before or after PBF [21,22,51,52]. However, in Cameroon, the government introduced subsidies for delivery kits simultaneously during the introduction of PBF [51], and OOP expenses for some family planning contraceptives and informal costs were reduced because of PBF [51,55]. In the Democratic Republic of Congo, providers considerably reduced OOP expenses for specific services [52].

In the pilot PBF study in Cameroon, PBF led to a significant reduction in household informal OOP expenses with a difference of US$3.64, as well as a significant reduction in laboratory and x-ray fees by US$2.38 and official provider fees for antenatal care by US$1.68 [51]. A cross-sectional study in Cameroon reported the use of PBF home visits to improve the utilization of modern family planning contraceptives by 59% vs 46% in the control group [55]. This was reported in an area where most of the population are farmers, with limited time to listen to family planning health promotion messages from the radio [55]. However, the authors did not report if there were any implications on cost, but rather reported that condoms were free at public health facilities, in addition to a subsidy for other modern family planning contraceptives in public facilities at an average cost of US$2.63; but they did not mention if this was due to PBF or not [55].

One RCT from the Democratic Republic of Congo assessed the percentage of paying for consultations and sending gifts to providers, but this was not disaggregated and may have included other forms of consultation not related to ANC, family planning, and/or skilled birth delivery [52]. However, the study reported a significant cost reduction for drugs [52] and noted that most facilities with user fees reduced due to PBF were those with initially high user fees [52]. Generally, user fees have been reported to be higher in private than in public facilities [19,48,51,52]. In Tanzania, the improved availability of drugs in turn minimized the need to pay for drugs in private pharmacies, which are usually expensive [19,45]. One RCT study from Cameroon reported on the availability of family planning contraceptives but did not report any effect associated with this on cost [51].

### Impact on outcomes: Direction of relative effect as rated in GRADE

We used the GRADE tool to assess the overall strength of the evidence with respect to effect estimates and reported results [36,37]. We present and briefly summarize the outcome details in the summary of findings table (Table 3) using EPOC’s recommended plain language summary approach [36] and the relative effect. Generally, the evidence is of low certainty. We noted that, PBF interventions with a standard comparator or standard care yielded positive results compared to when PBF was compared against other additional demand side enhancing interventions. A total of 10 studies met the eligibility criteria as described above for the GRADE assessment.

**Table 3:** Effect of PBF on OOP expenses to improve access and use of ANC, skilled birth delivery and family planning – GRADE summary of findings*

**Patients or population: Health care providers, pregnant women, health facilities, districts.**				
Settings: Sub-Saharan Africa-Cameroon, Tanzania, Burundi, Malawi, Burkina Faso.				
Intervention: Performance-based financing program.				
Comparison: Any comparator or standard practice.				
**Outcomes**	**Relative change**	**Number of studies**	**Certainty of evidence†**	**Comments**
Probability of paying OOP expenses during skilled birth delivery	Effect ranges from -31 to -42%	2 [19,45]	Moderate	PBF enforced exemption of OOP expenses for skilled birth delivery which had a positive effect on institutional delivery in two studies all from Tanzania. Sensitivity: No RCT measured or reported on this. Subgroup by wealth: one of the CBA study for which relative change could not be calculated for subgroup reported greater reduction in the probability of paying for deliveries amongst the poorest group relative to the least poor.
Use of skilled birth delivery in public vs private facilities	8.30%	3 [19,44,45]	Low	Two CBA studies from Tanzania reported increased in skilled birth delivery more in public facilities due to adherence to user fee exemption policy and improved availability of drugs which in turn minimizes the need to pay for drugs in private pharmacies. One CBA study from Burundi reported no change in public vs private facilities but did not provide details in the results. Sensitivity: No RCT measured or reported on this.
Use of skilled birth delivery (based on effect of OOP as a mediating factor)	9.4%	2 [19,45]	Moderate	Two CBA studies from Tanzania reported increased in skilled birth delivery due to the effect of PBF in enforcement of OOP expenses.
Use of skilled birth delivery (facility or institutional deliveries)	Effect ranges from 0.05% to 9.4%	10 [17,19,43-46,49-51,54]‡	Low	PBF probably have a positive effect on facility deliveries (based on five studies). The other studies that relative change could not be calculated reported a positive improvement in utilization rate (downgraded due to risk of bias issues with some studies) Sensitivity: two RCTs reported 0.05 to 5.6%. Excluding the two CBA studies from Tanzania, 0.05 to 5.6%. Subgroup analysis (four studies): studies with additional demand side incentives to offset OOP expenses relative change indicates -0.01% for one RCT study, and two CBA studies for which relative change could not be calculated reported increased use of institutional deliveries amongst women in the middle wealth quintile and uninsured households. Subgroup by wealth groups (five studies): for additional demand vs standard PBF by wealth quintile; lower 20% quintile, -0.10%; upper 80% -0.14% for one RCT study, and three CBA studies for which relative change could not be calculated reported increased use of institutional deliveries amongst women in the middle wealth quintile and uninsured households (downgraded due to risk of bias issues).
Caesarean section (EmOC)	N/A	1 [46]	Low	Only one study reported on this, and relative change could not be calculated.
Use of any ANC visits	Relative change ranges from 1.14 to 3.3%	4 [43,44,49,51]	Low	PBF may have little or no effect on the use of any ANC visit. One CBA study reported positive effect on overall ANC visits. Sensitivity: one RCT indicates 1.14%. No subgroup effect measured or reported for this category
Use of ANC in 1st trimester	0.08%	3 [44,50,54]‡	Low	PBF may have desirable effect. One RCT 0.08. One CBA for which relative change could not be calculated reported no change (suggested likely due to the high utilization rate of more than 1 ANC of up to 96%). Sensitivity analysis RCT indicates 0.08%. Subgroup analysis (three studies): with additional incentives to offset OOP and stimulate demand relative change indicates -0.1 for one RCT, other two studies relative change could not be calculated, one ITS reported greater improvement trend compared to control especially in health facilities when compared to hospitals, and one CBA for which relative change could not be calculated reported no change. Subgroup by wealth groups (two studies): relative change lower 20% -0.10%; upper 80% -0.14% from one RCT study and other study – CBA reported no change for poor vs non poor.
Use of four or more ANC visits	5.5% to -0.01%	3 [45,50,54]‡	Low	PBF may have desirable effect. one CBA no effect for the last two months as assessed. Sensitivity: one RCT indicates -0.01 to -0.06. Subgroup analysis (two studies): With additional demand side incentives to offset OOP expenses effect indicates -0.06%. Subgroup by wealth groups (one study): relative change lower 20%; 0.03%; upper 80%; -0.10% for one RCT (risk of bias concerns).
Use of family planning methods	0.18 to -1.80	6 [43,45,49-51,54]‡	Low	PBF may have desirable effects. Sensitivity assessment: two RCTs, one relative change 0.18 and one relative change could not be calculated reported increase in use of family planning relative to control data from facility register. Subgroup analysis (two studies); with additional demand side incentives to offset OOP expenses relative change indicates -0.19% for 1 RCT study (concerns with risk of bias). Subgroup by wealth quintile (one RCT study) relative change lower 20% -0.193%; upper 80%; -0.33%.
Family planning counselling on contraceptive options	N/A	1 [50]		Not possible to extract, only one study reported on this.

GRADE – Grading of Recommendations Assessment, Development and Evaluation, ANC – antenatal care, RCT – randomized controlled trial, OOP – out-of-pocket expense, CBA – controlled before-and-after, PBF – performance-based financing, N/A – not applicable, EmOC – emergency obstetric care

*Most studies had limitations on one or more aspects of risk of bias and indirectness (as most did not directly meet the patient/population, intervention, comparison, and outcome (PICO) assessment in terms of reporting the mediating effect or disaggregating data correctly) and inconsistency.

‡Definitions from EPOC Group below on the grading of the evidence. High – this research provides a very good indication of the likely effect. The likelihood that the effect will be substantially different is low. Moderate – this research provides a good indication of the likely effect. The likelihood that the effect will be substantially different is moderate. Low – this research provides some indication of the likely effect. However, the likelihood that it will be substantially different is high. Very low – this research does not provide a reliable indication of the likely effect. The likelihood that the effect will be substantially different is very high. Substantially different – a large enough difference that it might affect a decision [36].

‡The RCT study [54] that reported on subgroup for almost all indicators had concerns for risk of bias and reported contextual limitations and timing of data collection. Subgroup for additional demand-side incentives implies either conditional cash transfer support, community-based program exempting poor and/or user fee exemption policy for the poor or specific policy on user fee exemption following PBF implementation.

### Effect on skilled birth delivery

All 10 studies (from five countries) reported on skilled birth delivery. There was moderate evidence from two CBA studies from Tanzania that measured the probability of paying OOP expenses for skilled birth delivery, and relative effect indicates that PBF may decrease OOP expenses for skilled birth delivery by 31% to 42% (**Table 3**). The two studies from Tanzania indicate PBF’s relative effect on OOP expenses may probably improve skilled birth delivery by 9.4% in private and 8.3% in public facilites [19,45]. Low certainty evidence from three studies (two RCTs and one CBA) that measured the effect of PBF on skilled birth delivery without reporting the mediating role on OOP expenses indicate PBF may improve skilled birth delivery from as low as 0.05% to 5.6% [49,51,54]. Three CBA studies reported on PBF effect on skilled birth delivery in public facilities [19,44,45], while four other studies (two RCTs and one ITS) reported no significant change in institutional deliveries [49-52]. However, one ITS study from Malawi reported on a higher baseline utilization of skilled birth delivery of 90% [50]. A pilot CBA study in Burundi reported an increased percentage of institutional deliveries which was limited to selected provinces, but found no impact on this indicator at the national level [43,44]. With PBF additional equity-enhancing components as reported in one RCT study, it may slightly decrease utilization of skilled birth delivery as low as 0.009% [54].

### Effect on ANC

Almost all studies reported high baseline utilization rates for the first ANC visit, ranging between 78% and 96% with low certainty of evidence. Although PBF had little or no impact on this indicator, an observed increase in the quality of ANC provided was reported, represented by increased consumption of anti-tetanus vaccines [43,44,54], antimalarial drugs [19,45,50], and ion-based medications [45,54], which are all components related to ANC care. Four studies reported on the use of ANC visits; PBF may lead to little or no difference in ANC visits ranging from 1.14% to 3.3% [44,45,50,51]. Moreover, three studies reported on ANC visits in the 1st trimester, with little or no difference indicating a relative change of 0.08% or a slight reduction by 0.1% [44,50,54]. In one cluster randomized controlled trial (C-RCT) study, PBF with an additional enhancing financing mechanism slightly reduced the utilization of ANC in the 1st trimester by 0.1% [54], however, the study reported power limitations [54]. Three studies reported on the use of four or more ANC visits; they found that PBF may improve utilization by 5.5%, while possibly slightly reducing it in some contexts by 0.01% [45,50,54]. In one RCT study, PBF with additional enhancing financing mechanisms slightly reduced four or more ANC visits by 0.06% [54]. For the interrupted time series, the average monthly provision of ANC increased in Burkina Faso with the implementation of the nationwide free program [49]. No study reported on ANC visits in the second and/or third trimester. Rather, they focused more on the use of more than one ANC or any ANC, which does not help capture appropriate trends in utilization to monitor and understand the breakpoints and relevant gaps in access and utilization. One CBA study reported a greater effect on the probability of paying OOP expenses during ANC but a positive effect on the probability of giving gifts during ANC [45].

### Effect on family planning

Seven studies reported PBF effect on family planning with low certainty of evidence [43,45,47,49-51,54]. The relative effect of two studies (CBA and a C-RCT) indicated that PBF may improve utilization of family planning by 0.18%, while another study found that it may decrease utilization by 1.8% [45,54]. Another C-RCT study found that PBF with additional equity elements may have the relative effect of decreasing utilization of family planning by 0.19% [54] yet it also reported power limitations. Three other studies where the relative effect could not be calculated reported increased use of modern family planning [43,50,51] and one study reported no impact on family planning with limitations on power and the timing of data collection [50]. One study reported on family planning counselling [50]. A pilot study in Burundi reported an increase in the use of family planning which was limited to selected provinces, but found no impact on these indicators when the study was extended to the national level [43,44]. In Burkina Faso, the average monthly provision of family planning increased with the implementation of the nationwide free program [49].

### Impact on equity

Five [17,44,46,49,54] of the 10 assessed studies reported on the distributional effect of PBF by wealth group. All study countries (ie, Tanzania, Burkina Faso, Burundi, and Malawi) implemented some form of a user fee exemption intervention either as a national policy exemption, demand-side financing, or additional community-based insurance targeting poor individuals. All studies used household characteristics and asset ownership to categorize wealth groups into various quintiles. One CBA study further used the predisposing and enabling characteristics suggested by Andersen et al. to define the population groups [17]. However, none of the studies provided a definition of poor in the context of PBF vis-à-vis the context of the study in relation to the scale being used. One C-RCT study noted this as a limitation that the wealth grouping in their study did not match the characteristics of those targeted as poor individuals in the PBF community-based program [54].

A Tanzanian study reported increased utilization of institutional deliveries among women from the intermediate wealth quintile and uninsured households likely due to the increased adherence to user fee exemption in public facilities and the improved availability of drugs [17,19]. An indication of a pro-poor effect on the rate of institutional deliveries in public facilities was reported in Tanzania, with a change in health care cost amongst the poor and intermediate households and women in rural districts but not among the least poor [17,45]. Some limitations were reported in the statistical power and timing of data collection in one of the studies [17]. In Burundi, an increase in the proportion of institutional deliveries, especially among better-off individuals, was reported, but there was no effect on poor individuals and no changes in the place of residence were found [44].

In Burkina Faso, the relative effect observed in one C-RCT study on utilization of ANC in the first trimester had a slight improvement (0.10%) for the lowest wealth quintile and a slight decrease (0.14%) for the highest quintile [54]. There was a similar pattern for four or more ANC visits and a slight improvement (0.03%) in utilization for the lowest quintile and a decreased (0.10%) for the highest quintile [54]. With additional financing incentives, the study reported a slight decrease across all income groups in utilization for family planning with a relative effect of 0.19% for the lowest quintile and 0.33% for the highest quintile [54]. Following the abolishment of user fees in Burkina Faso, the Ministry of Health removed the additional payments for equity interventions that were targeted for certain groups of population and services [54]. The equity interventions did not generate additional benefits compared to PBF alone for women and those from the poorest category [49,54]. Some potential contextual limitations were reported in relation to inadequate human resources and structural constraints, delays in payments, and previous experience with poor implementation approaches [49,54]. For skilled birth delivery, there was a slight increase in utilization among the highest quintile (0.0007%) and a slight decrease for the lowest quintile (0.0001) [54]. The study reported some power and contextual limitations as the study data was collected about one year after the launch of the national free health care policy targeting women, which also influenced PBF equity measures as some additional compensations were abolished which may have also demotivated health workers from exploring innovative approaches to stimulate demand [49,54]. Similarly, in the Democratic Republic of Congo, abolishing additional compensation was reported to have demotivated workers and increased the rate of staff absenteeism [52].

In Burundi, there was no indication of the heterogeneity of effects for ANC and family planning by poverty status [43], no improvement in institutional deliveries, and no difference between public and private facilities [44]. However, an increase in institutional deliveries was reported among nonpoor individuals (wealthier group), but no effect was found among poor individuals [44]. The Burundi study also reported that although the user costs have been waived, the transportation costs may have likely affected the ability of poor women to deliver in a health facility [44].

## DISCUSSION

Generally, the evidence we found in our review is of low certainty. We identified that PBF as a stand-alone supply side strategy can motivate providers to reduce OOP expenses for certain maternal services, such as in Cameroon, and the Democratic Republic of Congo. However, the reduction may not stimulate demand to generate impact on utilization due to other contextual and individual demand-side barriers. In settings in which policies regarding free health care at the point of use have been instituted, such as Tanzania, or in which services have been subsidized, such as Burkina Faso, PBF likely facilitates or enables the utilization of skilled birth delivery. While the effective implementation of free health care policy or subsidized services may be hindered by operational barriers at the health facility or hidden costs, PBF may facilitate the removal of such operational barriers to enhance utilization of services especially in public facilities.

### Impact on access and utilization

Based on the earlier stated PBF assumption, PBF can be considered a behavioural change mechanism whereby providers are motivated with incentives through policy changes (contextual enabling characteristics) to stimulate demand by a change in cost (as one of the possible pathways), thereby increasing the ability of individuals to access services based on affordability (individual enabling characteristics) and quality of care [19,28]. According to the Andersen et al.’s framework, these contextual and individual enabling characteristics are a function of potential access, which may be either equitable or inequitable [28]. Thus, any PBF policy on OOP expenses is an instrument of potential access. However, these policy strategies are also approached differently by health facilities and health sectors to enhance the individual enabling characteristics towards improving access. These enabling characteristics thus translates to realized access, which is the actual utilization of services [28]. Most studies reported an improvement in the availability of drugs, which is an indicator of potential access in terms of an indirect potential reduction in OOP expenses for women who otherwise need to purchase drugs from the private sector, which is expensive [19]. In some countries, PBF favoured individuals from the middle-income and sometimes high-income quintiles, and in limited cases those from the low-income quintile. One question, however, is how this translates to effective access, which is a function of both potential and realized access.

In Tanzania, PBF was found to be effective in re-enforcing the user fee exemption policy and led to an immediate increase in the utilization of skilled birth delivery, with an even greater increase observed in public facilities [19,45]. In Malawi, PBF was found to be effective in improving access to ANC because of “resilience against interrupted supply chains and improvements in attendance rates” [50]. PBF was also found to be effective in the timing of ANC during the first trimester in some countries [^,^50,54], and for even more than four ANC visits in some contexts [44,50], but not among the poor quintile for more than four ANC visits in Burkina Faso [54]. In Burkina Faso and Burundi, an inequitable increase in utilization was observed among nonpoor individuals [44,49,54]. In the Democratic Republic of Congo, despite an increased in efforts by providers to reduce prices, PBF did not increase utilization, and the study suggests poor strategies employed by providers to stimulate demand given an existing poor level of satisfaction toward the quality of services, especially when most of the population are poorly educated and informed [52]. In Burkina Faso, poor implementation approaches and delay in payments were reported [49]. In Cameroon, the increased efforts made by providers to reduce user fees, including reductions in the prices of laboratory services and significant reductions in informal fees, among other efforts, did not lead to any significant change in the level of utilization for antenatal care and skilled birth delivery, suggesting existing high OOP expenses [51] ·

### Policy implications

The evidence on the effect of PBF as a way of reducing financial barriers by decreasing OOP expenses to improve access is low and of poor quality, especially when isolating the effect of PBF as a supply side strategy from other demand side strategies like conditional cash transfers and/or policy like removal of user fees.

PBF designs may appear similar, but implementation varies by context and contextual characteristics seem to play a major role in determining the outcome of the PBF. Based on the findings, PBF effect on equity was not significant both in settings with existing high OOP expenses and those with free or subsidized services, and/or combined implementation of CCT and supply. In some settings, there was no effect on equity. Therefore, it is important to carefully design PBF interventions to align with country specific problems and contexts and or assess other strategies that may generate the same results with significant effect on equity. OOP is an indicator for assessing equity, so all its elements should be incorporated to understand various equitable and inequitable process changes; if a policy is not impacting the poor or addressing equity concerns, it should be reviewed and re-strategized. Since PBF is an addition to existing payment mechanisms (with the mixed results of PBF effect from studies and possible administrative cost implications in the implementation), it is worth assessing its cost/benefit as a policy mechanism within specific context to enhance access to maternal health services. Most studies did not conduct such assessments, indicating an important research gap.

The PBF definition on equity aspects has not been well studied [56]; as reported in one of the studies, the classification of “poor” was not in line with the PBF community approach [54].There is a need to clearly define the “poor” within each context and align with the PBF definition of who constitute the poor, to ensure appropriate classification and reporting to inform policy intervention strategies towards achieving universal health coverage.

### Methodological considerations and study limitations

We observed some level of bias in all studies included in this review. There are substantial differences in the reporting of effect estimates, intervention designs, outcomes, and study groups, which precluded statistical pooling of the results. The effect estimates reported for PBF on OOP expenses for the public and private sectors were often absent; some studies rather controlled for it and did not use the opportunity to assess potential differences. Most importantly, PBF has been implemented in most countries over a reasonable period. In countries where some policy changes and adaption are ongoing [57], research must focus on designing and collecting new data to assess the policy over time and not focus only on data from the pilot studies which may have their own limitations. Moreover, most pilot studies are designed and conducted in an ideal situation, therefore, the findings may not reflect the actual implementation processes and challenges over time. This implies that there is limited evidence to inform relevant areas of the policy that may require revision or adoption to support advocacy and sustainability.

We assessed the quality of the review online using the AMSTAR tool and any disagreements were discussed to obtain consensus. The results from the two independent reviewers indicates moderate quality rating, which implies more than one non-critical weakness, but no critical flaws [39]. This means that the review may provide an accurate summary of the results of the included studies [39]. One weakness of this review, as per the AMSTAR tool, is the lack of a meta-analysis to enable an assessment of publication bias; furthermore, we did not assess funding sources from the individual sources [39].

One strength of this systematic review is its focus on sub-Saharan Africa where there is an interest in assessing the PBF-induced effect on OOP expenses to improve access and utilization of maternal services [30,31]. We initially aimed to update some outcomes of a published Cochrane review [58], but it had been updated and published during the review process [25]. However, some studies included here were not considered in the summary of findings of the Cochrane review, especially those studies published after 2018. The protocol was slightly modified at full text screening to exclude some of the papers that did not provide detailed information on OOP expenses but still met the PICO criteria; at this stage, we also excluded some of the papers that were included in the updated Cochrane review. Finally, the dimension of access should be interpreted with caution; it is focused specifically on discussing the PBF assumption on OOP expenses as used in this review and based on what was reported in the studies, which may not comprehensively reflect what is being implemented in the country.

## CONCLUSIONS

The results of our review are mixed, with important contextual differences and implementation approaches. Given that PBF is an addendum to an existing payment mechanism to stimulate behaviour change, the implementation of PBF can be considered a potential access instrument in reducing OOP expenses to stimulate demand for maternal services. However, the implementation approaches employed will determine utilization (realized access), taking into consideration existing equitable and inequitable access characteristics which vary by context.

## Additional material


Online Supplementary Document
