# Supplementary material for: A systematic review of the effect of performance-based financing interventions on out-of-pocket expenses to improve access to, and the utilization of, maternal health services across health sectors in sub-Saharan Africa
Source: J Glob Health. 2023 Apr 21;13:04035. doi: 10.7189/jogh.13.04035 (PMC10160705; doi:10.7189/jogh.13.04035)
Supplement: Online Supplementary Document [file jogh-13-04035-s001.pdf]

Supplemental File 1. Applying the Dimensions of access by Andersen at al., in the context PBF as a health financing policy

Dimensions of Access	Definition	Operationalization for purposes of data extraction in systematic review
Potential Access	Health financing policy in place in this context PBF	Implementation of performance-based financing policy
Realized Access	Actual utilization of services as a result of PBF policy	Utilization of antenatal care, skilled birth delivery due to PBF as reported in the study
Effective Access	Improving health status from health service use and is a function of potential and realized access	Changes in utilization as reported in the study as a result of the timely use of services in the implementation of the health financing policy
Equitable Access	distribution of resources based on need	Consideration of equity variables--income groups, place of residence and contextual differences in resource allocation as reported in the study
Inequitable Access	Focused on the process of reducing the influence of social characteristics on the distribution of health services	Consideration of approaches used to address information barriers amongst population groups as reported in the study
Efficient Access	To minimize the cost of improving outcomes as a result of health service use	Cost minimization for specific services (ANC, skilled birth delivery and/or family planning) to improve utilization and outcome

Source: The first two columns are based on Andersen et al., 2013 and the last column is the application approach in the context of the review

Supplemental File 2: A systematic review of the effect of performance-based financing interventions on out-of-pocket expenses to improve access to and the utilization of maternal health services in sub-Saharan Africa

SR Search strategy for PBF

Ovid Technologies, Inc. Email Service

Search for: 14 and 24

Database: Ovid MEDLINE(R) ALL <1946 to November 13, 2020>
Search Strategy:
----
1 africa/ or exp "africa south of the sahara"/ (237719)
2 (Afghanistan or Albania or Algeria or Angola or Argentina or Armenia or Armenian or Azerbaijan or Benin or Botswana or Burkina Faso or Burkina Fasso or Upper Volta or Burundi or Urundi or Cameroon or Cameroons or Cameron or Camerons or Cape Verde or Central African Republic or Chad or Comoros or Comoro Islands or Comores or Mayotte or Congo or Zaire or Cote d'Ivoire or Ivory Coast or Djibouti or French Somaliland or East Timur or Egypt or United Arab Republic or Eritrea or Ethiopia or Gabon or Gabonese Republic or Gambia or Ghana or Guinea or Kenya or Lesotho or Liberia or Libya or Macedonia or Madagascar or Malaya or Malawi or Mali or Mauritania or Mauritius or Mozambique or Niger or Nigeria or Rwanda or Ruanda or Sao Tome or Senegal or Seychelles or Sierra Leone or Somalia or Sudan or Swaziland or South Africa or Tanzania or Togo or Togolese Republic or Tonga or Uganda or western Sahara or West Bank or Zambia or Zimbabwe).ti,ab,kf. (379661)
3 1 or 2 (469877)
4 Reimbursement, Incentive/ or remuneration/ or "salaries and fringe benefits"/ (20260)
5 reimbursement.ti,ab,kf. (23402)
6 (pay* adj2 performance*).ti,ab,kf. (2510)
7 ((incentiv* or compensat* or reimburs*) adj2 plan?).ti,ab,kf. (771)
8 (conditional adj2 (pay* or transfer?)).ti,ab,kf. (432)
9 ((result* or perform* or output* or out put*) adj2 (financ* or fund* or pay* or disburs* or fee?)).ti,ab,kf. (11692)
10 ((pay* or monetar* or economic* or financ*) adj2 (compensation or incentive? or reward* or bonus or bonuses)).ti,ab,kf. (11182)
11 (financial support* or remunerat* or salar* or earnings).ti,ab,kf. (22775)
12 (performance adj2 financing*).ti,ab,kf. (213)
13 4 or 5 or 6 or 7 or 8 or 9 or 10 or 11 or 12 (81653)
14 3 and 13 (2551)

Ovid Technologies, Inc. Email Service

Search for: 14 and 24

Results: 50

Database: Embase Classic+Embase <1947 to 2020 November 18>
Search Strategy:
----
1 africa/ or exp "africa south of the sahara"/ (320875)
2 (Afghanistan or Albania or Algeria or Angola or Argentina or Armenia or Armenian or Azerbaijan or Benin or Botswana or Burkina Faso or Burkina Fasso or Upper Volta or Burundi or Urundi or Cameroon or Cameroons or Cameron or Camerons or Cape Verde or Central African Republic or Chad or Comoros or Comoro Islands or Comores or Mayotte or Congo or Zaire or Cote d'Ivoire or Ivory Coast or Djibouti or French Somaliland or East Timur or Egypt or United Arab Republic or Eritrea or Ethiopia or Gabon or Gabonese Republic or Gambia or Ghana or Guinea or Kenya or Lesotho or Liberia or Libya or Macedonia or Madagascar or Malaya or Malawi or Mali or Mauritania or Mauritius or Mozambique or Niger or Nigeria or Rwanda or Ruanda or Sao Tome or Senegal or Seychelles or Sierra Leone or Somalia or Sudan or Swaziland or South Africa or Tanzania or Togo or Togolese Republic or Tonga or Uganda or western Sahara or West Bank or Zambia or Zimbabwe).ti,ab,kw. (487843)
3 1 or 2 (596125)
4 reimbursement.mp. [mp=title, abstract, heading word, drug trade name, original title, device manufacturer, drug manufacturer, device trade name, keyword, floating subheading word, candidate term word] (70055)
5 reimbursement.ti,ab,kw. (36836)
6 (pay* adj2 performance*).ti,ab,kw. (3196)
7 ((incentiv* or compensat* or reimburs*) adj2 plan?).ti,ab,kw. (972)
8 (conditional adj2 (pay* or transfer?)).ti,ab,kw. (494)
9 ((result* or perform* or output* or out put*) adj2 (financ* or fund* or pay* or disburs* or fee?)).ti,ab,kw. (18319)
10 ((pay* or monetar* or economic* or financ*) adj2 (compensation or incentive? or reward* or bonus or bonuses)).ti,ab,kw. (14365)
11 (financial support* or remunerat* or salar* or earnings).ti,ab,kw. (30230)
12 (performance adj2 financing*).ti,ab,kw. (228)
13 4 or 5 or 6 or 7 or 8 or 9 or 10 or 11 or 12 (128033)
14 3 and 13 (3204)

Ovid Technologies, Inc. Email Service

Search for: 3 and 13

Results: 100

Database: EBM Reviews - Cochrane Central Register of Controlled Trials <October 2020>, EBM Reviews - Cochrane Database of Systematic Reviews <2005 to November 19, 2020>
Search Strategy:
----
1 africa/ or exp "africa south of the sahara"/ (6669)
2 (Afghanistan or Albania or Algeria or Angola or Argentina or Armenia or Armenian or Azerbaijan or Benin or Botswana or Burkina Faso or Burkina Fasso or Upper Volta or Burundi or Urundi or Cameroon or Cameroons or Cameron or Camerons or Cape Verde or Central African Republic or Chad or Comoros

or Comoro Islands or Comores or Mayotte or Congo or Zaire or Cote d'Ivoire or Ivory Coast or Djibouti or French Somaliland or East Timur or Egypt or United Arab Republic or Eritrea or Ethiopia or Gabon or Gabonese Republic or Gambia or Ghana or Guinea or Kenya or Lesotho or Liberia or Libya or Macedonia or Madagascar or Malaya or Malawi or Mali or Mauritania or Mauritius or Mozambique or Niger or Nigeria or Rwanda or Ruanda or Sao Tome or Senegal or Seychelles or Sierra Leone or Somalia or Sudan or Swaziland or South Africa or Tanzania or Togo or Togolese Republic or Tonga or Uganda or western Sahara or West Bank or Zambia or Zimbabwe).ti,ab,kw. (19650)
3 1 or 2 (21311)
4 reimbursement.mp. [mp=ti, ot, ab, sh, hw, kw, tx, ct] (1797)
5 reimbursement.ti,ab,kw. (1466)
6 (pay* adj2 performance*).ti,ab,kw. (154)
7 ((incentiv* or compensat* or reimburs*) adj2 plan?).ti,ab,kw. (30)
8 (conditional adj2 (pay* or transfer?)).ti,ab,kw. (179)
9 ((result* or perform* or output* or out put*) adj2 (financ* or fund* or pay* or disburs* or fee?)).ti,ab,kw. (1443)
10 ((pay* or monetar* or economic* or financ*) adj2 (compensation or incentive? or reward* or bonus or bonuses)).ti,ab,kw. (2010)
11 (financial support* or remunerat* or salar* or earnings).ti,ab,kw. (1105)
12 (performance adj2 financing*).ti,ab,kw. (21)
13 4 or 5 or 6 or 7 or 8 or 9 or 10 or 11 or 12 (6123)
14 3 and 13 (332)

CINAHL (EbscoHost)

#	Query	Limiters/Expanders	Results
S1	(MH "Africa South of the Sahara+")	Expanders - Apply related words; Apply equivalent subjects Search modes - Boolean/Phrase	69,364
S2	T1(Afghanistan or Albania or Algeria or Angola or Argentina or Armenia or Armenian or Azerbaijan or Benin or Botswana or Burkina Faso or Burkina Fasso or Upper Volta or Burundi or Urundi or Cameroon or Cameroons or Cameron or Camerons or Cape Verde or Central African Republic or Chad or Comoros or Comoro Islands or Comores or Mayotte or Congo or Zaire or Cote d'Ivoire or Ivory Coast or Djibouti or French Somaliland or East Timur or Egypt or United Arab Republic or Eritrea or Ethiopia or Gabon or Gabonese Republic or Gambia or Ghana or Guinea or Kenya or Lesotho or Liberia or Libya or Macedonia or Madagascar or Malaya or Malawi or Mali or Mauritania or Mauritius or Mozambique or Niger or Nigeria or Rwanda or Ruanda or Sao Tome or Senegal or Seychelles or Sierra Leone or Somalia or Sudan or Swaziland or South Africa or Tanzania or Togo or Togolese Republic or Tonga or Uganda or western Sahara or West Bank or Zambia or Zimbabwe)	Expanders - Apply related words; Apply equivalent subjects Search modes - Boolean/Phrase	4
S3	S1 OR S2	Expanders - Apply	69,367

		related words; Apply equivalent subjects Search modes - Boolean/Phrase	
S4	(MH "Physician Incentive Plans") OR (MH "Salaries and Fringe Benefits")	Expanders - Apply related words; Apply equivalent subjects Search modes - Boolean/Phrase	27524
S5	(MH "Fee for Service Plans") OR (MH "Reimbursement, Incentive")	Expanders - Apply related words; Apply equivalent subjects Search modes - Boolean/Phrase	5203
S6	TI reimbursement OR AB reimbursement	Expanders - Apply related words; Apply equivalent subjects Search modes - Boolean/Phrase	13115
S7	TI pay* N2 performance* OR AB pay* N2 performance*	Expanders - Apply related words; Apply equivalent subjects Search modes - Boolean/Phrase	2044
S8	TI (((incentiv* or compensat* or reimburs*) N2 plan?)) OR AB (((incentiv* or compensat* or reimburs*) N2 plan?))	Expanders - Apply related words; Apply equivalent subjects Search modes - Boolean/Phrase	324
S9	TI ((conditional N2 (pay* or transfer?))) OR AB ((conditional N2 (pay* or transfer?)))	Expanders - Apply related words; Apply equivalent subjects Search modes - Boolean/Phrase	227
S10	TI (((result* or perform* or output* or "out put*") N2 (financ* or fund* or pay* or disburs* or fee?))) OR AB (((result* or perform* or output* or "out put*") N2 (financ* or fund* or pay* or disburs* or fee?)))	Expanders - Apply related words; Apply equivalent subjects Search modes - Boolean/Phrase	6441
S11	TI (((pay* or monetar* or economic* or financ*) N2 (compensation or incentive? or reward* or bonus or bonuses))) OR AB (((pay* or monetar* or economic* or financ*) N2 (compensation or incentive? or reward* or bonus or bonuses)))	Expanders - Apply related words; Apply equivalent subjects Search modes - Boolean/Phrase	5426
S12	TI (("financial support*" or remunerat* or salar* or earnings)) OR AB (("financial support*" or remunerat* or salar* or earnings))	Expanders - Apply related words; Apply equivalent subjects Search modes - Boolean/Phrase	11173
S13	S4 OR S5 OR S6 OR S7 OR S8 OR S9 OR S10 OR S11	Expanders - Apply	61823

	OR S12	related words; Apply equivalent subjects<br>Search modes - Boolean/Phrase	
S14	S3 AND S13	Expanders - Apply related words; Apply equivalent subjects<br>Search modes - Boolean/Phrase	1041
S15	S3 AND S13	Expanders - Apply related words; Apply equivalent subjects<br>Search modes - Boolean/Phrase	1041

Supplemental file 3: Excluded Papers

Authors/Year/Country	Reasons for exclusion
1. Basinga et al., 2011, Rwanda	Did not provide sufficient information in relation to PBF on OOP to be included
2. Lannes et al.,2016, Rwanda	Did not provide sufficient information in relation to PBF on OOP to be included
3. Chansa et al., 2015, Zambia	Did not provide sufficient information in relation to PBF on OOP to be included
4. Chansa et al., 2020, Zambia	Did not provide sufficient information in relation to PBF on OOP to be included
5. Friedman et al 2016, Zambia	Did not provide sufficient information in relation to PBF on OOP to be included
6. Kreindrebeogoet al.,2015 Chad/Tchad	Did not provide sufficient information in relation to PBF on OOP to be included
7. Rudasingwa et al., 2017 Burundi	Did not provide sufficient information in relation to PBF on OOP to be included
8. Skiles et al.,2013, Rwanda	Did not provide sufficient information in relation to PBF on OOP to be included
9. Sato & Belel 2020 Nigeria-Adamawa state	Did not provide sufficient information in relation to PBF on OOP to be included
10. Steenland et al.,2017 Burkina Faso	Did not provide sufficient information in relation to PBF on OOP to be included
11. Zizen et al., 2019 Burkina Faso	Did not provide sufficient information in relation to PBF on OOP to be included
12. Zombre et al., 2020 Mali	Did not provide sufficient information in relation to PBF on OOP to be included
13. Zang et al2015, Cameroon	Did not provide sufficient information in relation to PBF on OOP to be included
14. Zeng et al., 2018 Republic of the Congo	Did not provide sufficient information in relation to PBF on OOP to be included
15. Basinga P,2009 Rwanda	Did not provide sufficient information in relation to PBF on OOP to be included
16. Soeters 2009	Did not provide sufficient information in relation to PBF on OOP to be included
17. Soeters et al.2005	Did not provide sufficient information in relation to PBF on OOP to be included
18. Shapira et al., 2018 Rwanda	Did not provide sufficient information in relation to PBF on OOP to be included
19. Vergeer 2008	Did not provide sufficient information in relation to PBF on OOP to be included
20. De Walque et al.,2018 Cameroon	Did not provide sufficient information in relation to PBF on OOP to be included
21. Brennar et al., 2018 Malawi	Did not meet PICO criteria for outcome measure
22. Brennar et al., 2017	Did not provide sufficient information in relation to PBF on OOP to be included
23. Binyaruka & Anselmi,2020	Did not provide sufficient information in relation to PBF on

	OOP to be included
24. Chansa et al., 2020 Zambia	Did not provide sufficient information in relation to PBF on OOP to be included
25. Chinkhumba et al., 2020 Malawi	Did not meet PICO criteria for outcome measure, focus on cost measures
26. De Allegri et al.,2019, Malawi	Did not provide sufficient information in relation to PBF on OOP to be included
27. De Allegri et al.,2019, Burkina Faso	Did not provide sufficient information in relation to PBF on OOP to be included
28. Falisse et al., 2015 Burundi	Did not provide sufficient information in relation to PBF on OOP to be included
29. Gage & Bauhoff,2021	Did not meet PICO
30. Jacobs et al.,2020 CAR, Nigeria and DRC	Did not meet PICO
31. Kiendrébéogo et al.,2015 Chad	Did not provide sufficient information in relation to PBF on OOP to be included
32. Mussah et al., 2017 Liberia	Did not meet PICO
33. Nahimana et al., 2016 Rwanda	Did not provide sufficient information in relation to PBF on OOP to be included
34. Paul et al.,2017 Benin	Did not meet PICO
35. Rajkotia et al., 2017 Mozambique	Did not provide sufficient information in relation to PBF on OOP to be included
36. Rusa et al.,2009 Rwanda	Did not meet PICO
37. Rudasingwa et al.,2015 Burundi	Did not provide sufficient information in relation to PBF on OOP to be included
38. Rudasingwa et al., 2014 Burundi	Did not provide sufficient information in relation to PBF on OOP to be included
39. Shapira et al., 2018	Did not meet PICO
40. Soeters et al.,2011 DRC	Did not provide sufficient information in relation to PBF on OOP to be included
41. Turcotte-Tremblay et al.,2018 Burkina Faso	Did not meet PICO
42. Mofoka et al., 2017 Lesotho	Did not provide sufficient information in relation to PBF on OOP to be included
43. Chukwuma et al., 2017 Nigeria	Did not provide sufficient information in relation to PBF on OOP to be included
44. Zeng et al., 2018 Zambia	Did not meet PICO elements
45. Rudasingwa et al., 2017 Burundi	Did not provide sufficient information in relation to PBF on OOP to be included
46. Beauge et al 2020 Burkina Faso	Did not meet PICO
47. Kandpal et al., 2018 Nigeria	Did not provide sufficient information in relation to PBF on OOP to be included
48. Mofoka et al., 2017 Lesotho	Did not meet PICO
49. Brennar et al., 2014 Malawi	Did not meet PICO

50. Sieleunou et al., 2020 Cameroon	Did not meet PICO
51. Oyebola et al., 2014 Nigeria	Did not meet PICO
52. Chinkhumba et al., 2015 Malawi	Did not provide sufficient information in relation to PBF on OOP to be included
53. Sekabaraga et al.,2011 Rwanda	Did not meet PICO
54. Borghi et al., 2013 Tanzania	Did not meet PICO
55. Zambia impact evaluation 2014	Did not provide sufficient information in relation to PBF on OOP to be included
56. Manthalu et al., 2016 Malawi	Did not meet PICO
57. Maini et al., 2014 Congo	Did not meet PICO
58. Ntambue et al., 2018 Congo	Did not meet PICO
59. Zeng et al., 2018 Zimbabwe	Did not meet PICO
60. Manzi et al., 2019 Rwanda	Did not meet PICO
61. Binyaruka et al., 2018 Tanzania	Did not provide sufficient information in relation to PBF on OOP to be included
62. Gotto et al., 2017 Uganda	Did not provide sufficient information in relation to PBF on OOP to be included
63. Rajkotia et al.,2015 Mozambique	Did not provide sufficient information in relation to PBF on OOP to be included
64. Bezu et al., 2021 Tanzania	Did not provide sufficient information in relation to PBF on OOP to be included
65. Binyaruka et al., 2017 Tanzania	Did not provide sufficient information in relation to PBF on OOP to be included
66. Mcmahon et al., 2016 Malawi	Did not meet PICO
67. Ngo et al., 2017	Did not meet PICO
68. Fox et al., 2014	Did not meet PICO
69. Nimpagaritse et al., 2016	Did not meet PICO
70. Janssen et al., 2015	Did not meet PICO
71. Grittner 2013	Did not meet PICO
72. Gergen et al., 2018	Did not meet PICO

Supplemental File 4: Risk of bias using EPOC criteria-Rated High, Unclear, Low

Within-study risk of bias in interrupted time series studies

Studies	Was the intervention independent of other changes?	Was the shape of the intervention effect pre-specified?	Was the intervention unlikely to affect data collection?	Was knowledge of the allocated interventions adequately prevented during the study	Were incomplete outcome data adequately addressed	Was the study free from selective outcome reporting	Was the study free from other risks of bias? (Cofounders and seasonality)
**Brenner et al., 2020**	Unclear (not specified)	Low (defined as per guidance)	Low (same data source)	Low	Unclear	Low (no indication)	Low (adjusted for seasonality and auto correlation
**Kunnibe et al., 2020**	Unclear (not specified)	Low (defined as per guidance)	Low (same data source)	Low	Low	Low (no indication)	Low (adjusted for cofounders, auto correlation)

Within-study risk of bias in controlled before-and-after studies

Study	Allocation sequence adequately generated?	Allocation adequately concealed?	Were baseline outcome measurements similar?	Were baseline characteristics similar?	Were incomplete outcome data adequately addressed?	knowledge of the allocated interventions adequately prevented during the study	Was the study adequately protected against contamination?	Was the study free from selective outcome reporting?	Was the study free from other risks of bias? (Adjusted for confounding, seasonality etc.?)
**Anselmi et al, 2017**	High (as per criteria)	High (as per criteria)	Low (analysis adjusted for potential	Low (comparable)	Unclear (not clearly specified)	Low (objective outcome)	Low (facility received intervention)	Low (no indication)	Low (controlled for confounders)

			differences)						
**Ashir et al., 2013**	High (as per criteria)	High (as per criteria)	High (as per criteria)	High (as per criteria)	Unclear (not specified)	Low (objective outcome)	Low (facility & community)	Unclear (not specified)	High (as per criteria)
**Binyaruka et al., 2018**	High (as per criteria)	High (as per criteria)	Low (analysis adjusted for potential differences)	Low(comparable)	High (as per criteria)	Low (objective outcome)	Low (facility received intervention)	Low (no indication)	Low (controlled for confounders)
**Bonfrer et al.,2014a**	High (as per criteria)	High (as per criteria)	Low(analysis adjusted for potential differences)	High (some differences in the districts)	Unclear	Low (objective outcome)	Low (facility received intervention)	Low (no indication)	Low (controlled for confounders and time variant
**Bonfrer et al.,2014b**	High (as per criteria)	High (as per criteria)	High (see table 3)- maybe low	Low(comparable)	Unclear	Low (objective)	Low (facility intervention)	Unclear (P-values no CI	low
**Binyaruka et al., 2015**	High (as per criteria)	High (as per criteria)	Low (analysis adjusted potential differences)	Low(comparable)	High (authors suggested it may have biased results)	Low (objective outcome)	Low (facility received intervention)	Low (no indication)	Low
**Canavan 2010**	High (as per criteria)	High (as per criteria)	High (as per criteria)	High (as per criteria)	Unclear	Low (objective outcome)	Unclear	Unclear	High (as per criteria)
**Chinkhumba et al.,2017**	High (as per criteria)	High (as per criteria)	Low	Low(comparable)	Low	Low (objective	Low (facility received	Low (no indication)	Low (controlled for

						outcome)	intervention)		confounders)
**Brenner et al., 2018**	High (as per criteria)	High (as per criteria)	Low (analysis approach)	High (as per criteria)	Low	Low (objective outcome)	Low (facility received intervention)	Low (no indication)	Low (controlled for potential confounders)

Within-study risk of bias in RCT studies

Study	Allocation sequence adequately generated	Allocation adequately concealed	Were baseline outcome measurements similar?	Were baseline characteristics similar	Were incomplete outcome data adequately addressed?	knowledge of the allocated interventions adequately prevented during the study	Was the study adequately protected against contamination?	Was the study free from selective outcome reporting?	Was the study free from other risks of bias? confounding, seasonality?
**De Walque et al.,2017**	Low(Public randomization)	Low (blinded)	Low(analysis adjusted for potential differences)	Low	Unclear	Low	Low	Unclear (inconsistency in reporting some outcomes	Low
**Ferguson et al., 2020**	Unclear	Unclear	Low	Low	Low	Low	Low	Unclear(P-value reported without CI)	Low
**Huillery & Seban, 2014**	Unclear	Unclear	Unclear	Unclear	Unclear	Low	Low	Unclear	Low

**Ferguson et al., 2022**	Unclear	Unclear	Low	Low	Low	Low	Low	Unclear	Low

Within-study risk of bias in Cluster RCT studies

**Study**	**Allocation sequence adequately generated**	**Allocation adequately concealed**	**Were baseline outcome measurements similar?**	**Were baseline characteristics similar**	**Were incomplete outcome data adequately addressed?**	**Knowledge of the allocated interventions adequately prevented during the study**	**Was the study adequately protected against contamination?**	**Was the study free from selective outcome reporting?**	**Was the study free from other risks of bias? confounding, seasonality?**
**Mwase et al., 2020**	Low	Low	High	Low	Unclear	Low	Low	Low	High (authors reported power limitation)
**Cross Sectional Study**									
**Study**	**Allocation sequence adequately generated?**	**Allocation adequately concealed?**	**Were baseline outcome measurements similar?**	**Were baseline characteristics similar?**	**Were incomplete outcome data adequately addressed?**	**knowledge of the allocated interventions adequately prevented during the study**	**Was the study adequately protected against contamination?**	**Was the study free from selective outcome reporting?**	**Was the study free from other risks of bias? (Adjusted for confounding, seasonality etc.?)**
**Egbe et al., 2016**	High (as per criteria)	High (as per criteria)	High	Low	Low	High (self reported)	High (authors reported potential contamination)	Low	Low (controlled for confounders)
